# ID 104 - Ezetimiba para o Tratamento de Indivíduos com Alto a Muito Alto Risco Cardiovascular: revisão sistemática com metaanálise de ensaios clínicos randomizados

**DOI:** 10.5327/2237-9622.2025.v34s1.104

**Published:** 2025-11-25

**Authors:** Tassiane Cristine Santos de Paula, Mariana Millan Fachi, Haliton Alves Oliveira, Rosa Camila Lucchetta, Layssa Andrade Oliveira

**Keywords:** ezetimiba, estatinas, doenças cardiovasculares, fatores de risco de doenças cardíacas, ensaios clínicos controlados aleatórios

## Abstract

**Introdução:**

As doenças cardiovasculares (DCV) são uma das principais causas de morbimortalidade globalmente. O tratamento da condição visa reduzir o colesterol LDL, sendo as estatinas a principal abordagem. No entanto, nem todos os pacientes atingem as metas com estatinas ou podem ainda apresentar intolerância às estatinas em altas doses. Nesse contexto, a combinação com ezetimiba pode ser uma opção, uma vez que age inibindo a absorção intestinal de colesterol. Nesse contexto, este estudo avalia a eficácia e a segurança da ezetimiba em indivíduos com alto a muito alto risco cardiovascular.

**Método:**

Foi realizada uma revisão sistemática buscando por ensaios clínicos randomizados (ECR) em PubMed, Embase e Cochrane Library que avaliaram ezetimiba combinada a estatinas (rosuvastatina, pravastatina, atorvastatina e sinvastatina) comparado as mesmas estatinas em monoterapia em pacientes de alto e muito alto risco cardiovascular. Os desfechos primários avaliados foram eventos adversos cardiovasculares maiores (MACE), incluindo morte por doença cardiovascular (DCV) e morte por outras causas, acidente vascular cerebral (AVC) não fatal e infarto agudo do miocárdio (IAM) não fatal. Já os desfechos secundários contemplaram a alteração do perfil lipídico (LDL-c, HDL-c e não HDL-c) e eventos adversos gerais e graves. O risco de viés e a qualidade da evidência foram avaliados pelo ROB 2.0 e GRADE, respectivamente. Metanálise diretas foram conduzidas para os desfechos primários e secundários.

**Resultados:**

Foram identificados 156 registros relacionados a 150 ECRs que avaliaram a combinação de ezetimiba com estatinas em comparação à monoterapia com estatinas, com tempo de acompanhamento médio de 56,8 semanas (dp: 14,6 semanas), sendo identificado que a associação de ezetimiba + estatinas demonstrou eficácia estatisticamente maior para os desfechos primários IAM (RR 0,88, IC 95% 0,81-0,95; GRADE: alta) e MACE (RR 0,93, IC 95% 0,89-0,97; GRADE: alta), e para os desfechos secundários de alteração do perfil lipídico (LDL-c: DM -0,10, IC 95% -0,12 a -0,09; colesterol total: DM -0,08, IC 95% -0,09 a -0,05; não-HDL-c: DM -0,12, IC 95% -0,14 a -0,10 e HDL-c: DM 0,01, IC 95% 0,01 a 0,04) quando comparado à estatina em monoterapia. Ainda, não foi identificada diferença entre as alternativas para os desfechos primários de eficácia AVC (RR 0,88, IC 95% 0,76 a 1,02; GRADE: moderada), morte por todas as causas (RR 0,99, IC 95% 0,92 a 1,07; GRADE: moderada) e morte por DCV (RR 1,00, IC 95% 0,89 a 1,12; GRADE: moderada) e para os desfechos de segurança (evento adverso grave: RR: 1,04, IC 95%: 0,86 a 1,25; evento adverso geral: RR 0,99, IC 95% 0,96 a 1,03 e descontinuação por EA: RR 1,06, IC 95% 0,98 a 1,14).

**Conclusão:**

Evidências clínicas demonstram que a combinação de ezetimiba + estatinas é preferível à monoterapia de estatinas em pacientes de alto e muito alto risco cardiovascular, com base em desfechos como MACE, IAM, alteração do perfil lipídico e segurança, sendo uma opção terapêutica para essa população. No entanto, não houve diferença estatisticamente significativa para mortalidade por todas as causas e eventos cardiovasculares maiores, o que ser explicado pelo reduzido tempo de acompanhamento dos estudos.

